# Tuberculosis Risk among Medical Trainees, Pune, India

**DOI:** 10.3201/eid2203.151673

**Published:** 2016-03

**Authors:** Anita Basavaraj, Ajay Chandanwale, Akhil Patil, Dileep Kadam, Samir Joshi, Nikhil Gupte, Katie McIntire, Divyashri Jain, Hamza Dalal, Rohan Badave, Andrea DeLuca, Amita Gupta, Robert Bollinger, Vidya Mave

**Affiliations:** Byramjee-Jeejeebhoy Medical College, Pune, India (A. Basavaraj, A. Chandanwale, A. Patil, D. Kadam, S. Joshi, H Dalal, R Badave);; Byramjee-Jeejeebhoy Medical College Clinical Trials Unit, Pune (A. Basavaraj, A. Chandanwale, D. Kadam, S. Joshi, N. Gupte, D. Jain, A. Gupta, R. Bollinger, V. Mave);; Johns Hopkins University School of Medicine, Baltimore, Maryland, USA (N. Gupte, K. McIntire, A. Gupta, R. Bollinger, V. Mave);; Johns Hopkins Bloomberg School of Public Health, Baltimore (A. Deluca)

**Keywords:** tuberculosis and other mycobacteria, healthcare workers, medical trainees, occupational diseases, India, respiratory infections, TB

## Abstract

During 2012–2013, at a public hospital in Pune, India, 26 (3.9%) cases of tuberculosis were reported among 662 medical trainees, representing an estimated incidence of 3,279 cases/100,000 person-years. Three of these infections were isoniazid-resistant, 1 was multidrug-resistant, and 1 occurred in a trainee who had fulminant hepatitis after starting treatment for TB.

India has the world’s largest burden of tuberculosis (TB) and multidrug-resistant (MDR) TB; estimated community TB incidence is 167 cases/100,000 person-years (*1*,*2*). Occupational TB risk is elevated worldwide; however, limited available data suggest that healthcare workers (HCWs), specifically medical trainees, may be at particularly high risk in India and other countries with high TB incidence (*3*–*5*). Although the World Health Organization (WHO) has long recommended infection control guidelines for TB prevention among HCWs, implementation globally has been suboptimal (*6*). To prioritize improved local implementation of WHO guidelines, documenting TB risk among HCWs and medical trainees is urgently needed. We estimated TB prevalence and TB incidence and investigated the frequency of key TB treatment outcomes among medical trainees at a public teaching hospital in India.

## The Study

During June 2012–December 2013, we conducted a retrospective study among HCWs at Byramjee-Jeejeebhoy Government Medical College–Sassoon General Hospital (BJGMC-SGH), a 1,300-bed public teaching hospital in Pune in the state of Maharashtra, India. All major clinical and preclinical departments were made aware of the study objectives and referred HCWs who self-identified as having TB to the study team. After obtaining written consent, we entered demographic and clinical data from HCW interviews and clinical and laboratory data from medical records into case report forms. Medical trainees were defined as medical residents or interns.

Routine sputum acid-fast bacilli (AFB) smear and culture were performed at baseline for suspected pulmonary TB (PTB) and repeated at 2 months and, if positive, at 3 months and at the end of treatment. Participants with suspected extrapulmonary TB (EPTB) (e.g., pleural TB, meningitis, or lymphadenitis) underwent additional AFB smear, culture, or histopathologic evaluation of the affected sites as appropriate. All AFB cultures were performed on Lowenstein-Jenson media. Drug-susceptibility testing was performed routinely on all positive sputum and EPTB cultures at baseline and at each follow-up evaluation by using the proportion method (*7*). Routine HIV testing was performed under the national program (*2*). The BJGMC-SGH Institutional Ethics Committee approved all study methods.

Primary end points included estimated TB prevalence and TB incidence per 100,000 person-years among medical trainees. To estimate prevalence and incidence, we obtained the denominator (the total number of medical trainees) from employment records. TB prevalence was calculated by dividing the number of TB cases by the total number of medical trainees. Estimated incidence was calculated as the number of TB cases multiplied by 100,000 and divided by the duration of exposure and the total number of medical trainees. Duration of exposure was 18 months for medical residents and 12 months for interns.

Secondary end points included cure (smear- or culture-negative at the end of treatment), treatment failure (smear- or culture-positive at month 5 or later), death (of any cause during treatment), and treatment success (cured and completed treatment) (*1*). We also evaluated positive AFB smear or cultures at month 2 of treatment, resistance to any anti-TB drug, MDR TB, and adverse events. MDR TB was defined as resistance to at least isoniazid and rifampin (*3*). Descriptive statistics were used to measure secondary outcomes. All analyses were conducted by using Stata version 10 (Stata Corp LP, College Station, TX, USA).

Among the 1,886 HCWs assessed in the study, 47 cases of TB were identified (*8*); 26 cases (14 in residents and 12 in interns) were identified among 662 medical trainees, who had 793 person-years of follow-up. Overall among medical trainees, TB prevalence was 3.9%, and estimated TB incidence was 3,279 cases/100,000 person-years (Table). The median age of trainees with TB was 27 (interquartile range 26–28) years; 14 (54%) were male, 9 (35%) had PTB, 17 (65%) had EPTB (6 [35%] with microbiological confirmation), and 17 (65%) had presumptive TB. Most worked in the general medicine (46%) or radiology (15%) departments (Figure). All trainees with TB were HIV negative.

**Table:** Estimated prevalence and incidence of TB among medical trainees at Byramjee-Jeejeebhoy Government Medical College–Sassoon General Hospital, Pune, India, June 2011–December 2013*

Category	All medical trainees, N = 662	Residents, n = 262	Interns, n = 400
No. TB cases (prevalence†)	26 (3.9)	14 (5.3)	12 (3.0)
Total person-years of exposure	793	393	400
Estimated TB incidence per 100,000 person-years‡ (95% Poisson CI)	3,279 (2,142–4,804)	3,562 (1,948–5,977)	3,000 (1,550–5,240)


*TB, tuberculosis. †Number of TB cases divided by the total number of medical trainees. ‡Number of TB cases multiplied by 100,000 and divided by the duration of exposure and the total number of medical trainees.

**Figure:**
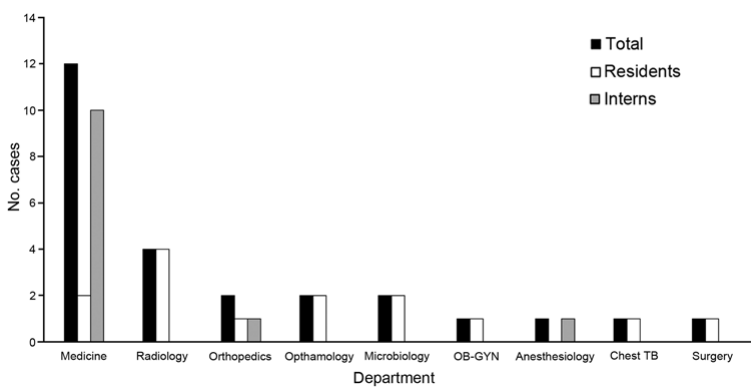
Number of TB cases among medical trainees, by department, at Byramjee-Jeejeebhoy Government Medical College–Sassoon General Hospital, Pune, India, June 2011–December 2013. TB, tuberculosis; OB-GYN, obstetrics–gynecology.

The frequency of MDR TB was 44% (4/9 cases of culture-positive TB); 3 trainees had TB with single-drug resistance to isoniazid and had their disease successfully treated with first-line anti-TB drugs, and 1 had MDR TB that was successfully treated with second-line anti-TB drugs. All other trainees with TB had their disease successfully treated with first-line anti-TB drugs, except 1 who experienced acute fulminant hepatic failure during TB treatment and required liver transplant and treatment with levofloxacin, ethambutol, and streptomycin. The frequency of adverse drug reactions was 62% (16/26), including 14 cases of gastritis. No cases of treatment failure or death were reported.

## Conclusions

Our investigation found a 15-fold higher estimated incidence of TB among medical trainees at BJGMC-SGH (3,279 cases/100,000 person-years) than among that reported for the community in the study state of Maharashtra, India (167 cases/100,000 person-years) (*2*). Although our findings are consistent with those of previous studies showing substantially elevated risk among medical trainees, we demonstrate ≈2-fold higher TB incidence among medical trainees than that indicated by a decade-old study of medical trainees from India (*5*,*6*); this increase is likely attributable to increasing TB incidence in the community. Increased TB risk among medical trainees is probably a function of duration of exposure. Another study from India reported a 4-fold higher prevalence of TB infection among older medical students (i.e., >23 years of age) compared with younger medical students (18–20 years of age) (*9*). Two notable findings in our study are that approximately two thirds of medical trainees had EPTB diagnosed and that approximately one third of those infections were microbiologically confirmed; these findings have also been observed in previous studies of HCWs in India (*4*,*5*).

Our study provides critical evidence that medical trainees in India are at risk for drug-susceptible and drug-resistant TB, including TB with single-drug resistance to isoniazid and MDR TB. MDR TB cases are probably underreported, and only 1 case of extremely drug-resistant TB in a HCW has been reported in India (*10*). With increasing MDR TB prevalence and the frequent need for hospitalization of these patients, the incidence of MDR TB among medical trainees in India may increase over time unless awareness is increased and improved infection control measures are rapidly implemented (*1*).

The consequences of active TB among medical trainees may have a substantial public health impact. First, medical trainees may be reluctant to seek early medical care because of stigma, fear of losing training time, poor knowledge, or a perceived lack of vulnerability to TB (*6*,*11*). Thus, trainees can secondarily transmit TB to peers in overcrowded hostels (most trainees reside in hostels close to teaching hospitals in India), patients in clinics and wards, and family members (*6*,*12*). Second, as observed in our study and others, a higher risk for adverse events exists among medical trainees (*4*,*5*). Trainees in whom major adverse drug reactions develop may choose to leave the profession permanently, as we observed with the trainee in whom acute fulminant hepatic failure developed during treatment for TB. Last, although routine treatment of TB and MDR TB is available to medical trainees, while not observed in our cohort, less than two thirds of patients with MDR TB experience successful treatment (*1*).

Our study has some limitations. We may have underestimated TB incidence because some medical trainees may not have reported incident TB because of stigma and unwillingness to participate in a study. In addition, we may have had some recall bias because of the retrospective nature of the study. Despite these limitations, our study underscores the immediate need to for education and implementation of infection control measures to safeguard medical trainees from TB (*6*,*7*). Additional large prospective studies are needed to evaluate the risk for latent TB infection and TB disease among medical trainees and the effectiveness of infection control measures.
